# Chronic Pain and Late-Life Depression are Positively Associated in Chinese Centenarians and Oldest-Old Adults

**DOI:** 10.1155/da/5565953

**Published:** 2025-08-28

**Authors:** Shihui Fu, Youchen Zhang, Kaifei Wang, Wenjun Lei, Qiong Liu, Jinwen Tian, Bo Li, Tianyang Yun, Yali Zhao, Jiacai Lin, Yunqi Li, Long Feng

**Affiliations:** ^1^Department of Cardiology, Hainan Hospital of Chinese PLA General Hospital, Hainan Geriatric Disease Clinical Medical Research Center, Hainan Branch of China Geriatric Disease Clinical Research Center, Sanya, Hainan, China; ^2^Department of Geriatric Cardiology, Chinese PLA General Hospital, Beijing, China; ^3^The Second School of Clinical Medicine, Southern Medical University, Guangzhou, Guangdong, China; ^4^Department of Pediatrics, Hainan Hospital of Chinese PLA General Hospital, Sanya, Hainan, China; ^5^College of Pulmonary and Critical Care Medicine, Chinese PLA General Hospital, Beijing, China; ^6^Department of Nephrology, Haikou People's Hospital, Haikou, Hainan, China; ^7^Medical Care Center, Hainan Hospital of Chinese PLA General Hospital, Sanya, Hainan, China; ^8^Department of Thoracic Surgery, Hainan Hospital of Chinese PLA General Hospital, Sanya, Hainan, China; ^9^Central Laboratory, Hainan Hospital of Chinese PLA General Hospital, Sanya, Hainan, China; ^10^Department of Neurology, Hainan Hospital of Chinese PLA General Hospital, Sanya, Hainan, China; ^11^Department of Gastroenterology, The First Medical Center of Chinese PLA General Hospital, Beijing, China; ^12^Department of Anesthesiology, Hainan Hospital of Chinese PLA General Hospital, Sanya, Hainan, China

**Keywords:** centenarians, chronic pain, geriatric depression scale, late-life depression, oldest-old

## Abstract

**Objectives:** Aging is an inevitable process. Chronic pain and late-life depression frequently coexist in older adults. This study was aimed to explore the association between chronic pain and late-life depression in Chinese centenarians and oldest-old adults.

**Study Design:** According to the list provided by the Department of Civil Affairs, a household survey was conducted on all centenarian and oldest-old adults residing in 18 cities and counties of Hainan Province.

**Methods:** The household survey method was used to collect basic information with interview questionnaires, physical examinations, and blood tests conducted by systematically trained doctors and nurses. This study used visual analog scales and numerical rating scale for pain assessment. Geriatric depression scale (GDS) was used for the evaluation of depression.

**Results:** All 1324 older adults had a median age of 91 years, ranging from 80 to 116 years. Among them, 349 older adults (26.4%) have depression, and 507 (38.3%) suffer from chronic pain. Comorbidity rate of chronic pain and late-life depression was 12.6% (167 participants). Furthermore, late-life depression (odds ratio [OR]: 1.591, 95% confidence interval [CI]: 1.218–2.078, and *p*=0.001) was significantly and positively associated with chronic pain in multivariate logistic regression analysis. Chronic pain (OR: 1.581, 95% CI: 1.210–2.067, and *p*=0.001) was significant and positive factor associated with late-life depression in multivariate logistic regression analysis.

**Conclusions:** This study demonstrated that chronic pain and late-life depression are positively associated in Chinese centenarians and oldest-old adults. This suggests that the management of pain should be considered when treating late-life depression in older adults.

## 1. Background

Late-life depression is a behavioral health condition in older adults. This mental health problem is characterized by disturbed appetite and sleep, persistent sadness, fatigue and inattention, and loss of interest or fun in previously beneficial or pleasant activities [1]. The prevalence of depression is 5.3% in the United States and 4.3% in China [2–5]. The combined prevalence of prolonged depression and its symptoms among Chinese bereaved family members was 8.9% and 32.4%, respectively [6]. A questionnaire survey was conducted among 752 older patients aged over 65 years in 13 community health service centers in Wuhan, China. One-fifth (20.3%) of these older adults met the depression in the month prior to the interview [7]. Recognition rate of depression in older patients was only 1.3%. Previous studies have found that depression was closely connected to severe medical problems in older adults [8]. Depression not only affects people with chronic diseases, but also can show chronic pain [9]. Therefore, in view of the growing older groups, it is recommended to strengthen depression management of these special groups.

The population-based studies have indicated that the prevalence of chronic pain in older adults was 55% for the population over 60 years, and up to 60% of older adults over 75 years [10]. According to the demographic epidemiological survey, the chronic pain rate in Canada was 15.1%, while that in Sweden was as high as 48.9% [11, 12]. In addition, the result of relevant studies in Asia has shown that chronic pain had a prevalence of 15.2% in Malaysia [13]. Chronic pain accounts for 40% of all symptom-related consultations in the United States, costing over $100 billion annually in medical costs and lost productivity. Chronic pain not only affects physical functions, but also deteriorates the quality of life in older adults [14, 15]. In a cross-sectional survey with a health check-up population in China, chronic pain has a prevalence of 11.0% [16]. The prospective cohort and nested case–control study using a nationally representative sample of twins aged 70 years and above pointed out that depression was associated with an increased probability of pain and could predict the occurrence and development of pain [17].

Aging is an inevitable process. Chronic pain and late-life depression frequently coexist in older adults [18]. Comorbidity rate of pain and depression is 6.9%–13% in older adults [19]. Bair et al. [18] have reported that the prevalence of depression in older adults with chronic pain ranged from 4.7% to 22% in population-based study and from 5.9% to 46% in primary care studies. A Chinese community survey has found that older women aged 60–79 years in China were more likely to experience depression from chronic pain [20]. Age is a key modified factor affecting the association between chronic pain and late-life depression [21, 22]. Unfortunately, there are limited researches reporting the prevalence and association of chronic pain and late-life depression in Chinese centenarians and oldest-old adults [23]. Centenarian and oldest-old adults are a special group with an increased age. Therefore, this full sample survey was aimed to explore the association between chronic pain and late-life depression among centenarians and oldest-old adults in China.

## 2. Materials and Methods

### 2.1. Study Population

This is an observational study with full sample survey of centenarian and oldest-old population in Hainan Province, China. According to the list of centenarian and oldest-old adults provided by the Department of Civil Affairs of Hainan Province, a household survey was conducted on all centenarian and oldest-old adults residing in 18 cities and counties of Hainan Province from June 2014 to December 2016. A survey sample of 1863 cases included 966 centenarians aged over 100 years and 897 oldest-old adults aged 80–99 years. Among them, 397 centenarians and 142 oldest-old adults had missing variables. So there were 569 centenarians and 755 oldest-old adults aged 80–99 years included in this study. This study was conducted in accordance with the Declaration of Helsinki and approved by the Medical Ethics Committee of Hainan Hospital of Chinese PLA General Hospital (301HNLL-2016-01). All participants provided written informed consent before participating in the study.

### 2.2. Household Survey

The household survey method was used to collect basic information with interview questionnaires, physical examinations, and blood tests conducted by systematically trained doctors and nurses. The covariates in this study included age, sex, body mass index, race, living, working, smoking, drinking, systolic blood pressure, diastolic blood pressure, heart rate, and analysis biomarkers. Administrator, professional, sales, and service personnel were categorized as mental work. Farmer, fisherman, herdsman, and laborer were categorized as manual work. Smoking was defined as the consumption of one or more cigarets per day for at least 1 year. Drinking was defined as the consumption of 30 g or more alcohols per week for at least 1 year. Blood samples were collected and transported in chilled biotransport containers (4°C) to the central laboratory within 4 h. Analysis biomarkers included red blood cell, hemoglobin, white blood cell, neutrophil, uric acid, creatinine, urea nitrogen, alanine aminotransferase, aspartate aminotransferase, total protein, and albumin analyzed with enzymatic analyses (Roche Products Ltd., Basel, Switzerland) on a fully automatic biochemical autoanalyzer (Cobas c702; Roche Products Ltd., Basel, Switzerland). Qualified technicians who performed laboratory analyses were blinded to clinical data.

### 2.3. Pain Assessment

This study used visual analog scales and numerical rating scale for pain assessment. Visual analog scales correspond to the expression: 0 point (no pain): the expression is relaxed and there is no discomfort; 1–3 points (mild pain): mild discomfort that does not interfere with daily activities, such as mosquito bites or minor abrasions; 4–6 points (moderate pain): significant pain that may interfere with sleep or activities, such as toothache or sprain; 7–8 points (severe pain): persistent and severe pain, accompanied by sweating and shortness of breath, such as pain from a fracture or after surgery; 9–10 points (extremely severe pain): unbearable pain that may be accompanied by syncope or shock, such as pain from severe trauma or advanced cancer. Numerical rating scale: 0 point: no pain; 10 points: the most severe pain (worse than death); mild (1–3 points): does not interfere with sleep, such as pain when turning over or coughing; moderate (4–6 points): persistent pain while lying still, affecting sleep; severe (7–10 points): pain leading to the inability to sleep or excessive sweating.

### 2.4. Depression Evaluation

Geriatric depression scale (GDS-15) has been proved to be an effective scale for the evaluation of depression older patients [24]. It is well known that the 15-item version of GDS-15 has been validated as an evaluating tool for depression in older people [25]. A cut-off point of GDS-15 ≥5 has a pooled sensitivity of 88% and specificity 64% for evaluating late-life depression in older people [26, 27]. Participants were regarded as late-life depression with a cut-off point of GDS-15 ≥5. All participants were inquired if they had chronic pain lasting more than 1 month by specialist physicians who could communicate in the local language.

## 3. Statistical Analyses

Statistical analyses were performed by Statistic Package for Social Science 19.0 software package (Chicago, IL, USA). Data were described using medians and interquartile ranges (continuous variables with skewed distributions) and numbers and percentages (categorical variables). Characteristic comparison was performed between participants with and without chronic pain and late-life depression using Mann–Whitney *U* tests for continuous variables with skewed distributions and Chi-square tests for categorical variables. Kolmogorov–Smirnov and Shapiro–Wilk tests were performed to assume their normality. Multivariate logistic regression analyses were performed to determine the factors associated with late-life depression and chronic pain after adjusting age, sex, body mass index, race, living, working, smoking, drinking, systolic blood pressure, diastolic blood pressure, heart rate, red blood cell, hemoglobin, white blood cell, neutrophil, uric acid, creatinine, urea nitrogen, alanine aminotransferase, aspartate aminotransferase, total protein, and albumin. A significant *p* level of 0.05 for two-sided test was used to test each hypothesis.

## 4. Results

**Figure 1:**
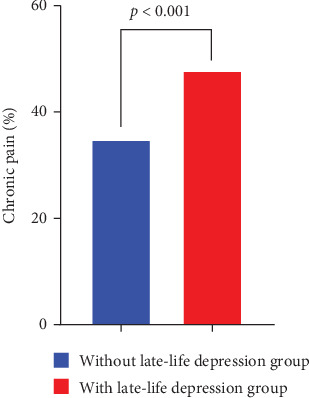
Comorbidity rate between chronic pain and late-life depression in Chinese centenarians and oldest-old adults: proportions of chronic pain with and without late-life depression compared with Chi-square tests (*p*  < 0.001).

All 1324 older adults had a median age of 91 years, ranging from 80 to 116 years. Males accounted for 31.3% (414 participants). Late-life depression was diagnosed in 349 (26.4%) of 1324 older adults. As shown in Table 1, age, sex, body mass index, proportions of chronic pain (Figure 1) and mental worker, and levels of red blood cell, hemoglobin, urine acid, creatinine, alanine aminotransferase, aspartate aminotransferase, total protein, and albumin had statistically significant difference between participants with and without late-life depression (*p*  < 0.05 for all). There were 507 participants (38.3%) with chronic pain in the 1324 older adults. As shown in Table 2, there were statistically significant difference in age, sex, and proportion of late-life depression between participants with and without chronic pain (*p*  < 0.05 for all). Comorbidity rate of chronic pain and late-life depression was 12.6% (167 participants). Late-life depression (odds ratio [OR]: 1.591, 95% confidence interval [CI]: 1.218–2.078, and *p*=0.001) was significantly and positively associated with chronic pain in multivariate logistic regression analysis Table 3. Chronic pain (OR: 1.581, 95% CI: 1.210–2.067, and *p*=0.001) was significant and positive factor associated with late-life depression in multivariate logistic regression analysis (Table 4).

## 5. Discussion

The World Health Organization (WHO) claims that by 2030, depression is one of the world's most common diseases [28]. This study showed that late-life depression and chronic pain had the prevalence of 26.4% and 38.3%, respectively, in Chinese centenarians and oldest-old adults. The latest study has shown that the prevalence of late-life depression was found to be 12.2% in Chinese rural older adults [29]. Another study has shown that the prevalence of depression is 36.9% in Chinese rural older adults [30]. Besides, two meta-analysis has also found that the prevalence of late-life depression was 19.47% in Western countries [31]. The results of our study showed that the prevalence of depression was higher in older adults than that in the general population and that provided by WHO worldwide [32]. The mainly reason responsible for this result is all study participants equal to or more than 80 years in our study. Similarly, recent data have shown that the prevalence of late-life depression was 29% in four European regions [33]. It is well known that the prevalence of depression increases with age, which not only severely affects health condition of older adults, but also increases family burden and reduces the quality of life [34–36].

Depression and pain are intertwined and coexist in nearly 80% of patients [34]. In these patients, psychological and social functions are reduced, and physical and mental symptoms are aggravated, resulting in high mortality [37, 38]. The prevalence of chronic pain in patients treated for depression ranges from 51.8% to 59.1%, whereas the prevalence of depression ranges from 18% to 85% in patients with chronic pain [39–41]. Our results indicated that chronic pain was related to late-life depression in Chinese centenarians and oldest-old adults. A national survey targeting centenarians in Denmark, which is similar to this result, showed that 21% of centenarians have experienced chronic pain, and depression was associated with more prevalent pain [22]. Besides, also researches have shown that there was a significant and positive association between pain and depression, and pain increased the risk for depression between 2.5 and 4.1 times [42, 43]. When controlling for covariates, pain remained being significantly associated with depression in the whole cohort. Horackova et al. [33] have shown that late-life depression had the strongest association with chronic pain. Bierman [44] has also proved that pain was positively related to depression among older adults. It is believed that the association between pain and depression is bidirectional, and over time, increased pain is likely to result in the occurrence and development of depression, and vise versa [45–47].

The mechanisms underlying the association between late-life depression and chronic pain are as follows: first, neuroinflammation plays a key role in the pathogenesis of late-life depression and chronic pain [8]; second, the important biochemical basis between late-life depression and chronic pain focuses on serotonergic and noradrenergic systems. Ketamine and cannabinoids appear to be safe and effective options for improving severe depression and pain [35]; third, central nervous system undergoing long-term plastic changes is also associated with late-life depression and chronic pain [41]; finally, the experiences of pain and depression share common genetic factors and biological pathways, indicating that genetic and physiological factors may make individuals more susceptible to pain and depression [18].

The symptoms of depression in later years may not appear depressive and sad emotions. On the contrary, they exhibit loneliness, weakness, cognitive impairment, suicidal ideation, loss of interest, and unexplained physical complaints [48]. Pain and depression in later years are associated with poor quality of life and higher mortality rates [49]. Related studies have found that older adults with pain can still respond to antidepressant treatment, and pain may be associated with more difficulty to treat depression [50]. In clinical practice, serotonin norepinephrine reuptake inhibitors are often used in combination with opioid drugs to treat chronic pain. Opioid drugs are negatively correlated with the early analgesic response of older adults to low-dose venlafaxine [51]. Alternative antidepressants can be chosen for pain management in complex patients [52, 53]. In the future, antidepressants should also be used to prevent and control chronic pain.

One of the most significant strengths of this study is the large sample of Chinese centenarians. Centenarian and oldest-old adults are a special group with an increased age representing aging as an inevitable process, and there are limited researches reporting the association between chronic pain and late-life depression in centenarians and oldest-old adults. However, this study has several limitations: first, the sample is geographically restricted to Hainan Province, potentially limiting the generalizability to other Chinese populations; second, the reliance on self-reported data for pain and depression may introduce recall bias, especially among older adults who may have cognitive impairments; third, this study does not differentiate between pain types (nociceptive pain vs. neuropathic pain) or assess the severity of depression beyond a binary cutoff, which could obscure nuanced association; fourth, it lacks data on potential mediating factors (inflammation markers and socioeconomic status), limiting mechanistic insights into observed relationships.

## 6. Conclusions

This study demonstrated that chronic pain and late-life depression are positively associated in Chinese centenarians and oldest-old adults. This suggests that the management of pain should be considered when treating late-life depression in older adults. It is essential to increase social awareness and develop health policies of early prevention and appropriate management of chronic pain and late-life depression to better enhance the quality of life and improve long-term prognosis of older adults in their later years.

## Figures and Tables

**Table 1:** Characteristics of participants with and without late-life depression.

Characteristics	Without late-life depression (*n* = 975)	With late-life depression (*n* = 349)	*X* ^2^/*Z*	*p*
Age (year)^a^	88 (83, 101)	101 (87, 103)	−8.030	<0.001
Males, *n* (%)^b^	353 (36.2)	61 (17.5)	41.937	<0.001
Body mass index (kg/m^2^)^a^	20 (17, 22)	19 (16, 21)	−5.659	<0.001
Chronic pain, *n* (%)^b^	340 (34.9)	167 (47.9)	18.322	<0.001
Race, *n* (%)^b^	—	—	0.944	0.815
Han	863 (88.5)	304 (87.1)	—	—
Li	108 (11.1)	44 (12.6)	—	—
Other	4 (0.4)	1 (0.3)	—	—
Living alone, *n* (%)^b^	159 (16.3)	69 (19.8)	2.162	0.141
Mental worker, *n* (%)^b^	42 (4.3)	3 (0.9)	9.307	0.002
Smoker, *n* (%)^b^	104 (10.7)	26 (7.4)	3.003	0.083
Drinker, *n* (%)^b^	136 (13.9)	44 (12.6)	0.394	0.530
Systolic blood pressure (mmHg)^a^	148 (133, 168)	150 (132, 168)	−0.415	0.652
Diastolic blood pressure (mmHg)^a^	77 (70, 86)	77 (68, 86)	−1.464	0.143
Heart rate (beats/min)^a^	77 (70, 86)	78 (70, 86)	−0.024	0.981
Red blood cell (10^9^/L)^a^	4.29 (3.91, 4.69)	4.04 (3.65, 4.47)	−6.572	<0.001
Hemoglobin (g/L)^a^	123 (113, 134)	117 (104, 126)	−7.094	<0.001
White blood cell (10^9^/L)^a^	6.06 (5.01, 7.32)	6.00 (5.00, 7.37)	−0.166	0.868
Neutrophil (10^9^/L)^a^	0.55 (0.48, 0.63)	0.57 (0.50, 0.63)	−1.588	0.112
Urine acid (μmol/L)^a^	334 (276, 397)	317 (259, 389)	−2.703	0.007
Creatinine (μmol/L)^a^	81 (67, 100)	78 (65, 94)	−2.141	0.032
Urine nitrogen (mmol/L)^a^	5.6 (4.5, 6.9)	5.6 (4.3, 7.2)	−0.587	0.557
Alanine aminotransferase (U/L)^a^	11.6 (9.0, 15.5)	10.0 (7.6, 13.0)	−6.707	<0.001
Aspartate aminotransferase (U/L)^a^	22.3 (19.2, 26.3)	21.0 (18.0, 24.7)	−4.444	<0.001
Total protein (g/L)^a^	71.1 (67.9, 74.9)	70.0 (65.2, 73.5)	−4.127	<0.001
Albumin (g/L)^a^	41.4 (39.0, 43.5)	39.7 (37.0, 42.5)	−6.482	<0.001

^a^Described using medians and interquartile ranges and compared with Mann–Whitney *U* tests.

^b^Described using numbers and percentages and compared with Chi-square tests.

**Table 2:** Characteristics of participants with and without chronic pain.

Characteristics	Without chronic pain (*n* = 817)	With chronic pain (*n* = 507)	*X* ^2^/*Z*	*p*
Age (year)^a^	90 (83, 102)	93 (84, 102)	−2.309	0.021
Males, *n* (%)^b^	288 (35.3)	126 (24.9)	15.742	<0.001
Body mass index (kg/m^2^)^a^	19 (17, 22)	20 (17, 22)	−1.245	0.213
Late-life depression, *n* (%)^b^	182 (22.3)	167 (32.9)	18.322	<0.001
Race, *n* (%)^b^	—	—	4.150	0.246
Han	718 (87.9)	449 (88.6)	—	—
Li	95 (11.6)	57 (11.2)	—	—
Other	4 (0.5)	1 (0.2)	—	—
Living alone, *n* (%)^b^	137 (16.8)	91 (17.9)	0.306	0.580
Mental worker, *n* (%)^b^	31 (3.8)	14 (2.8)	1.017	0.313
Smoker, *n* (%)^b^	85 (10.4)	45 (8.9)	0.825	0.364
Drinker, *n* (%)^b^	111 (13.6)	69 (13.6)	0.000	0.990
Systolic blood pressure (mmHg)^a^	148 (133, 168)	151 (132, 169)	−1.101	0.271
Diastolic blood pressure (mmHg)^a^	77 (70, 86)	77 (69, 87)	−0.461	0.645
Heart rate (beats/min)^a^	77 (70, 85)	78 (71, 86)	−1.635	0.102
Red blood cell (10^9^/L)^a^	4.27 (3.87, 4.67)	4.20 (3.82, 4.57)	−1.670	0.095
Hemoglobin (g/L)^a^	122 (112, 134)	120 (110, 131)	−2.349	0.091
White blood cell (10^9^/L)^a^	5.99 (4.96, 7.16)	6.09 (5.11, 7.46)	−1.948	0.051
Neutrophil (10^9^/L)^a^	0.56 (0.49, 0.63)	0.56 (0.48, 0.63)	−0.248	0.804
Urine acid (μmol/L)^a^	333 (275, 393)	326 (265, 397)	−1.078	0.281
Creatinine (μmol/L)^a^	81 (68, 100)	79 (65, 98)	−1.933	0.053
Urine nitrogen (mmol/L)^a^	5.5 (4.4, 6.8)	5.8 (4.5, 7.2)	−1.789	0.074
Alanine aminotransferase (U/L)^a^	11.0 (8.6, 14.2)	11.3 (8.8, 15.3)	−1.527	0.127
Aspartate aminotransferase (U/L)^a^	22.0 (18.8, 25.7)	21.8 (19.1, 25.9)	−0.671	0.502
Total protein (g/L)^a^	71.0 (67.4, 74.6)	70.6 (66.7, 74.3)	−1.785	0.074
Albumin (g/L)^a^	41.0 (38.2, 43.3)	41.0 (38.5, 43.2)	−0.083	0.934

^a^Described using medians and interquartile ranges and compared with Mann–Whitney *U* tests.

^b^Described using numbers and percentages and compared with Chi-square tests.

**Table 3:** Factors associated with chronic pain in multivariate logistic regression analysis.

Characteristics	Odds ratio	95% Confidence interval	*p*
Age (year)	1.009	0.994–1.025	0.232
Sex, *n* (%)	1.720	1.223–2.419	0.002
Body mass index (kg/m^2^)	1.038	1.002–1.076	0.037
Late-life depression, *n* (%)^a^	1.591	1.218–2.078	0.001
Race, *n* (%)	0.839	0.585–1.204	0.341
Living alone, *n* (%)	1.054	0.776–1.431	0.739
Mental worker, *n* (%)	0.984	0.494–1.956	0.962
Smoker, *n* (%)	1.088	0.711–1.666	0.698
Drinker, *n* (%)	1.347	0.936–1.938	0.109
Systolic blood pressure (mmHg)	1.000	0.994–1.006	0.910
Diastolic blood pressure (mmHg)	1.001	0.990–1.013	0.837
Heart rate (beats/min)	1.005	0.994–1.015	0.370
Red blood cell (10^9^/L)	1.026	0.790–1.332	0.848
Hemoglobin (g/L)	0.993	0.984–1.003	0.180
White blood cell (10^9^/L)	1.083	1.011–1.161	0.024
Neutrophil (10^9^/L)	0.568	0.208–1.554	0.271
Urine acid (μmol/L)	0.999	0.998–1.001	0.480
Creatinine (μmol/L)	1.001	0.996–1.006	0.765
Urine nitrogen (mmol/L)	1.020	0.968–1.076	0.456
Alanine aminotransferase (U/L)	1.023	0.998–1.049	0.072
Aspartate aminotransferase (U/L)	1.000	0.979–1.022	0.973
Total protein (g/L)	0.973	0.950–0.998	0.031
Albumin (g/L)	1.033	0.993–1.074	0.106

^a^Multivariate logistic regression analyses were performed to determine whether late-life depression was associated with chronic pain after adjusting age, sex, body mass index, race, living, working, smoking, drinking, systolic blood pressure, diastolic blood pressure, heart rate, red blood cell, hemoglobin, white blood cell, neutrophil, uric acid, creatinine, urea nitrogen, alanine aminotransferase, aspartate aminotransferase, total protein, and albumin.

**Table 4:** Factors associated with late-life depression in multivariate logistic regression analysis.

Characteristics	Odds ratio	95% Confidence interval	*p*
Age (year)	1.040	1.022–1.058	<0.001
Sex, *n* (%)	1.971	1.307–2.972	0.001
Body mass index (kg/m^2^)	0.976	0.938–1.016	0.238
Chronic pain, *n* (%)^a^	1.581	1.210–2.067	0.001
Race, *n* (%)	1.116	0.757–1.664	0.579
Living alone, *n* (%)	1.389	0.986–1.956	0.060
Mental worker, *n* (%)	2.038	0.600–6.926	0.254
Smoker, *n* (%)	1.132	0.670–1.904	0.644
Drinker, *n* (%)	1.249	0.816–1.911	0.307
Systolic blood pressure (mmHg)	0.994	0.998–1.001	0.091
Diastolic blood pressure (mmHg)	1.020	1.007–1.033	0.003
Heart rate (beats/min)	0.999	0.988–1.011	0.909
Red blood cell (10^9^/L)	0.809	0.597–1.097	0.173
Hemoglobin (g/L)	0.993	0.982–1.005	0.254
White blood cell (10^9^/L)	1.055	0.977–1.138	0.171
Neutrophil (10^9^/L)	1.021	0.530–1.967	0.951
Urine acid (μmol/L)	1.000	0.998–1.001	0.694
Creatinine (μmol/L)	1.001	0.996–1.007	0.641
Urine nitrogen (mmol/L)	0.973	0.913–1.036	0.385
Alanine aminotransferase (U/L)	0.995	0.963–1.028	0.771
Aspartate aminotransferase (U/L)	0.984	0.957–1.011	0.241
Total protein (g/L)	0.997	0.970–1.025	0.836
Albumin (g/L)	0.959	0.917–1.002	0.062

^a^Multivariate logistic regression analyses were performed to determine whether chronic pain was associated with late-life depression after adjusting age, sex, body mass index, race, living, working, smoking, drinking, systolic blood pressure, diastolic blood pressure, heart rate, red blood cell, hemoglobin, white blood cell, neutrophil, uric acid, creatinine, urea nitrogen, alanine aminotransferase, aspartate aminotransferase, total protein, and albumin.

## Data Availability

Data used in this study will be available upon request from the authors.
